# Maize stover mulching combined with an optimized fertilization strategy reshapes rhizosphere microbial communities and functions in greenhouse potato

**DOI:** 10.3389/fmicb.2026.1670904

**Published:** 2026-05-05

**Authors:** Baoqi Yuan, Chuang Li, Qingfeng Wang, Qi Yao, Xiaowei Guo, Zhongwei Wang

**Affiliations:** Jilin Academy of Agricultural Sciences (Northeast Agricultural Research Center of China), Changchun, China

**Keywords:** fertilization strategy, maize stover mulching, potato, protected cultivation, rhizosphere metagenomics

## Abstract

Protected cultivation systems offer opportunities for improving potato productivity but are often constrained by inefficient maize stover utilization and suboptimal fertilization practices. In this study, a 4 × 4 factorial experiment was conducted using the potato cultivar ‘Jishu No. 1’ to decode the rhizosphere microbial mechanisms underpinning plant growth and yield enhancement under greenhouse conditions. We hypothesized that integrated management (the synergy between stover mulching and fertilization) would modify the soil microenvironment, thereby reshaping microbial community assembly patterns and functional gene distributions. The results showed that while split fertilization combined with moderate stover mulching (F2S2, 8,500 kg·hm^−2^ stover mulching) was most effective in enhancing plant physiological status, full topdressing combined with the same mulching level (F3S2) achieved the highest agronomic productivity, increasing total yield to 42.33 t·hm^−2^. Metagenomic analysis revealed that the F3S2 strategy significantly reshaped the rhizosphere microbiome, characterized by higher α-diversity and the enrichment of pathways related to carbon metabolism and carbohydrate processing. Notably, F3S2 promoted the recruitment of copiotrophic taxa, particularly Actinobacteriota, whose relative abundance was significantly and positively correlated with soil organic phosphorus (*r* = 0.623, *p* < 0.05). In contrast, oligotrophic groups like Acidobacteriota were relatively less abundant in nutrient-rich treatments. These findings demonstrate that moderate stover mulching combined with dynamic fertilization provides a high-resource niche that favors functional microbial groups, thereby driving rhizosphere nutrient cycling to support potato performance. This study underscores the importance of optimized stover and fertilizer management strategies in protected cultivation.

## 1 Introduction

Potato (*Solanum tuberosum* L.) is among the most widely cultivated tuber crops globally, with an annual production exceeding 380 million tons (Zaheer and Akhtar, 2016). In northern China, protected cultivation systems have enabled intensive land use and off-season potato production, yet these systems often suffer from suboptimal agronomic practices, such as over-fertilization and declining soil biological quality (Visser et al., 2009; Zhang et al., 2017).

In Jilin Province, Northeast China—a major maize-producing region—over 40 million tons of stover are generated annually, most of which remain underutilized (Zhu et al., 2021). Meanwhile, large areas of idle rice seedling greenhouses represent untapped potential for sustainable off-season potato cultivation (Tein et al., 2014; Sha et al., 2022). Integrating stover resources through mulching offers a sustainable strategy to improve soil structure, conserve moisture, and enhance crop productivity (Fan et al., 2024). When combined with rational fertilization, these practices can exert synergistic effects not only on crop growth but also on rhizosphere microbial dynamics (Lian et al., 2023).

Soil microbial communities are increasingly recognized as critical regulators of plant health and productivity. They participate in nutrient cycling, hormone biosynthesis, and immune modulation at the root–soil interface (Du et al., 2022). Agronomic practices such as stover mulching and fertilization can significantly alter microbial composition and function (Akhtar et al., 2019; Chen et al., 2021). However, how these practices jointly influence the diversity, structure, and ecological stability of rhizosphere microbiota—particularly under protected cultivation conditions—remains insufficiently understood.

Recent advances in metagenomics offer powerful tools to investigate soil microbial diversity and metabolic capacity at high resolution (Trabelsi and Mhamdi, 2013). Applying these approaches in the context of integrated management practices provides an opportunity to uncover microbiome-mediated mechanisms that underpin soil–plant interactions and agronomic outcomes (Kunin et al., 2008; Wooley et al., 2010).

We hypothesized that the synergy between moderate stover mulching and optimized fertilization would effectively stabilize the rhizosphere microenvironment. To test this hypothesis, we established a 4 × 4 factorial experiment combining different stover mulching levels and fertilization regimes in greenhouse-grown potato (cv. Jishu No. 1). By integrating assessments of plant performance, soil physicochemical traits, and high-throughput metagenomic sequencing. Our objectives were to characterize the responses of microbial taxonomic structure and functional gene profiles to management combinations.

## 2 Materials and methods

### 2.1 Experimental site and materials

This study was conducted in a solar greenhouse at the Fanjiatun Experimental Station of the Institute of Economic Plants, Jilin Academy of Agricultural Sciences. The experimental site is located in Gongzhuling, Jilin Province, China (124°02′–125°18′E, 43°11′–44°09′N), which has a temperate humid continental monsoon climate, with an average annual temperature of approximately 5.6 °C and a frost-free period of 125–140 days. The greenhouse is an arched plastic structure, measuring 55 m in length and 12 m in width. The soil inside the greenhouse is classified as a Typic Black soil (Mollisols in the USDA Soil Taxonomy), with a loam texture, uniform structure, and good drainage.

The test variety was *Solanum tuberosum* ‘Jishu No. 1’, developed through a cross between ‘Neishu No. 7’ (female parent) and ‘Zaodabai’ (male parent), followed by systematic selection. The variety was officially registered in 2011 by Jilin Province and is characterized by early maturity, high commercial tuber ratio, yellow skin and flesh, and uniform tuber shape, making it suitable for both greenhouse and open-field cultivation. The tubers contain 24.62% dry matter, 18.91% starch, 0.19% reducing sugars, 6.58 mg/100 g vitamin C, and 2.54% crude protein. It exhibits strong resistance to late blight and moderate resistance to potato virus Y (PVY).

The mulching material used was crushed maize stover (including leaves, stalks, and cobs after grain removal), with particle lengths ≤30 cm. Three types of fertilizers were applied: (1) a compound fertilizer (N–P₂O₅–K₂O = 12:18:15, total nutrient content ≥45%) used as basal fertilizer; (2) monoammonium phosphate (12:61:0) applied as topdressing; and (3) potassium sulfate (K₂O ≥ 52%, S ≥ 17.5%, Cl ≤ 1%) as a potassium supplement (Ma et al., 2024). All fertilizers were pre-weighed and applied uniformly. Detailed pure nutrient inputs (N, P₂O₅, and K₂O) for each fertilization intensity are provided in Supplementary Table S2 to ensure comparability across fertilizer types (Supplementary Table S2).

### 2.2 Experimental design and treatment setup

**Figure 1:**
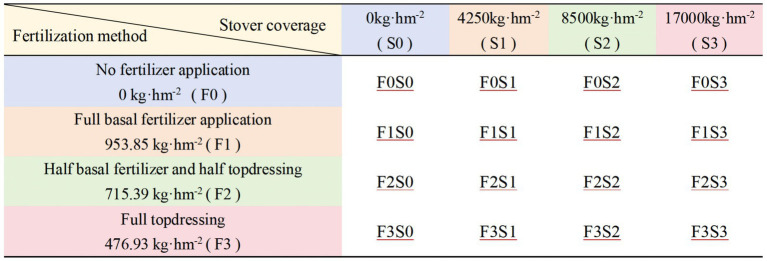
Experimental design combining fertilization regimes and stover mulching levels. The experiment employed a two-factor randomized block design with four fertilization treatments—no fertilizer application (F0, 0 kg·hm^−2^), full basal application (F1, 953.85 kg·hm^−2^), half basal plus half topdressing (F2, 715.39 kg·hm^−2^), and full topdressing (F3, 476.93 kg·hm^−2^)—in combination with four stover mulching levels: no mulching (S0), 4,250 kg·hm^−2^ (S1), 8,500 kg·hm^−2^ (S2), and 17,000 kg·hm^−2^ (S3). A total of 16 treatment combinations were established, each labeled as “F × S.” Three biological replicates were set per treatment.

A two-factor randomized complete block design was employed, with fertilization mode (F) and stover mulching rate (S) as the two main effect factors (Harbur et al., 2023). Each factor had four levels, resulting in a total of 16 treatment combinations, with three biological replicates per treatment (Figure 1; Supplementary Table S1). Each plot consisted of five ridges (ridge length: 10 m, width: 60 cm) with 20 cm plant spacing, covering an effective area of 30 m^2^. A 1 m buffer zone was set between plots to minimize cross-treatment interference.

The fertilization treatments represented different nutrient intensities and application timings: F0 no fertilizer (control); F1 (high intensity at 953.85 kg·hm^−2^, 100% basal); F2 (medium intensity, split application at 476.93 kg·hm^−2^ basal + 238.46 kg·hm^−2^ topdressing); and F3 (low intensity, 100% topdressing at 476.93 kg·hm^−2^). Please refer to Supplementary Table S1 for the specific pure nutrient amounts associated with each intensity. The stover mulching rates were defined as follows: S0: no mulching (control); S1: low mulching (4,250 kg·hm^−2^); S2: moderate mulching (8,500 kg·hm^−2^); S3: high mulching (17,000 kg·hm^−2^).

Treatment combinations were denoted as F × S (e.g., F1S2 = full basal fertilization + 8,500 kg·hm^−2^ stover mulching). All treatments were sown on May 28, 2023, using virus-free seed tubers of ‘Jishu No. 1’. Fertilizer application and stover mulching were completed prior to sowing, while topdressing was applied according to the crop growth stages. Field management followed standardized high-yield potato cultivation practices to ensure consistency and minimize interference from non-experimental factors.

### 2.3 Measurement of agronomic traits and soil physicochemical properties

#### 2.3.1 Photosynthetic characteristics and chlorophyll fluorescence parameters

Photosynthetic parameters including SPAD value, net photosynthetic rate (Pn), transpiration rate (Tr), stomatal conductance (Gs), and intercellular CO₂ concentration (Ci) were measured (Dahal et al., 2023). SPAD values were recorded using a SPAD-502 portable chlorophyll meter, while Pn, Tr, Gs, and Ci were measured using a LI-6400XT portable photosynthesis system (LI-COR Biosciences, USA). Measurements were conducted at the fourth fully expanded leaf on the main stem during the seedling, tuber initiation, starch accumulation, and maturation stages, between 9:00 and 11:00 a.m. on sunny days. The light intensity was set at 1,000 μmol·m^−2^·s^−1^, with CO₂ concentration maintained at approximately 380 μmol·mol^−1^.

Chlorophyll fluorescence parameters, including the maximum photochemical efficiency (Fv/Fm), actual quantum yield of PSII (Y(II)), photochemical quenching coefficient (qP), and non-photochemical quenching coefficient (NPQ), were measured using an OS5p + pulse-modulated chlorophyll fluorometer (Opti-Sciences, USA). Prior to measurement, leaves were dark-adapted for 30 min (Jan et al., 2024).

#### 2.3.2 Soil physicochemical properties

Soil samples were collected from the 0–20 cm topsoil layer during the tuber bulking stage (Wang Y. et al., 2023). After air-drying and sieving, samples were used for physicochemical analysis. Soil organic matter was determined using the potassium dichromate oxidation-external heating method. Hydrolyzable nitrogen was measured by the alkali hydrolysis diffusion method. Available potassium was extracted with 1 mol·L^−1^ ammonium acetate and determined by flame photometry. Available phosphorus was extracted with 0.5 mol·L^−1^ NaHCO₃ and quantified by the molybdenum antimony colorimetric method.

#### 2.3.3 Antioxidant enzyme activities and lipid peroxidation products

Physiological indicators in leaf samples were measured, including superoxide dismutase (SOD), peroxidase (POD), catalase (CAT) activities, and malondialdehyde (MDA) content (Abd El-Moneim et al., 2012). SOD activity was determined based on its inhibition of nitroblue tetrazolium (NBT) photoreduction. POD activity was measured using the guaiacol method. CAT activity was determined by monitoring the rate of H₂O₂ decomposition. MDA content was assessed by the thiobarbituric acid (TBA) colorimetric method. All assays were conducted at 4 °C, and absorbance was read using a UV–Vis spectrophotometer (UV-2600, Shimadzu, Japan).

#### 2.3.4 Tuber quality traits

Tuber quality traits included starch, reducing sugar, soluble protein, and vitamin C contents. Starch content was measured using the acid hydrolysis method. Reducing sugar content was determined by the 3,5-dinitrosalicylic acid (DNS) colorimetric method. Soluble protein content was measured using the Coomassie Brilliant Blue G-250 binding assay. VC content was determined by titration with 2,6-dichlorophenol-indophenol (Ahmad et al., 2022).

#### 2.3.5 Yield-related traits

At maturity, five representative plants were randomly selected from each plot. Plant height was measured from the base to the topmost growing point using a ruler, and stem diameter was measured at the base using a vernier caliper. The number and weight of tubers per plant were recorded using an electronic balance. Total yield per plot was calculated and converted to yield per hectare.

### 2.4 Principal component analysis

To comprehensively evaluate the combined effects of different stover mulching rates and fertilization strategies on potato yield and tuber quality, principal component analysis (PCA) was conducted to reduce the dimensionality of 21 measured traits (Placide et al., 2015). These traits encompassed photosynthetic parameters, soil physicochemical properties, yield-related traits, and quality indicators. Prior to analysis, all variables were standardized to eliminate the influence of differing units and scales. PCA was performed based on the covariance matrix, and principal components with eigenvalues greater than 1 were retained for further analysis. Each treatment combination was assigned scores along the selected principal components, and comprehensive scores were calculated accordingly. The treatments were then ranked based on their overall performance to support an integrated evaluation of treatment performance across multiple agronomic, physiological, and microbiome-related indicators. All statistical analyses and graphical visualizations were conducted using GraphPad Prism 9.0 (Mavrevski et al., 2018).

### 2.5 Metagenomic sequencing and bioinformatic analysis

Rhizosphere soil samples for metagenomic analysis were collected at the maturity stage to evaluate the cumulative effect of stover mulching and fertilization on the soil microbial legacy. The F3 series was prioritized for sequencing as it was identified as the optimal fertilization framework based on preliminary PCA rankings. Soil samples (0–20 cm) were collected from four representative treatments with three biological replicates each. These treatments were selected to represent contrasting fertilization strategies under different stover mulching levels within the same fertilization category, allowing microbial comparisons under a controlled fertilization framework. Total DNA was extracted using the FastDNA® Spin Kit for Soil, and sequencing was performed on the Illumina NovaSeq platform (paired-end 150 bp) (Bolger et al., 2014).

Quality control was conducted using KneadData (Langmead and Salzberg, 2012). Clean reads were assembled using MEGAHIT, and gene prediction was performed with Prodigal (Segata et al., 2011). A non-redundant gene catalog was generated via CD-HIT, and gene abundance was estimated using Salmon. Protein sequences were annotated against major functional databases (KEGG, eggNOG, GO, CAZy, CARD) using DIAMOND (Franzosa et al., 2018). Taxonomic classification was performed using Kraken2 and Bracken, complemented by BASTA-based annotation (Wood and Salzberg, 2014; Lu et al., 2017).

Alpha and beta diversity metrics were calculated using QIIME2 (Le Chatelier et al., 2013; Jia et al., 2016). Principal coordinate analysis (PCoA), LEfSe (LDA > 2.0), and functional enrichment analysis were conducted to identify key taxonomic and functional differences among treatments (Villar et al., 2015; Mandal et al., 2015). To provide a comprehensive assessment of the environmental biosafety and ecological stability of the stover-amended system, antibiotic resistance gene (ARG) profiles were further examined. Visualizations were created using R software.

### 2.6 Statistical analysis

All experimental data, including agronomic traits, physiological indices, tuber quality, and soil physicochemical properties, were expressed as mean ± standard deviation (SD) of three biological replicates. Statistical significance was evaluated by one-way ANOVA using SPSS Statistics 25.0 (IBM Corp., Armonk, NY, USA), with multiple comparisons performed using the LSD method at *p* < 0.05. Data visualization and annotation of significant differences were performed using GraphPad Prism 9.0 and R software.

## 3 Results

### 3.1 Phenotypic performance and soil nutrient dynamics as functional indicators

#### 3.1.1 Microclimate modulation under protected cultivation

**Figure 2:**
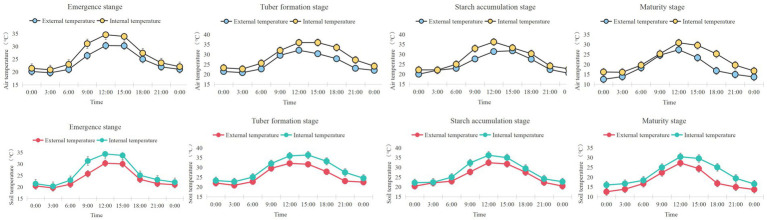
Diurnal variations in air and soil temperatures inside and outside the facility during four potato growth stages. Temperature data were recorded at four key growth stages—emergence, tuber formation, starch accumulation, and maturity. The upper row shows changes in air temperature (external vs. internal), while the lower row shows soil temperature fluctuations.

To investigate the influence of greenhouse environmental conditions on the physiological responses of potato, diurnal variations in air and soil temperatures were continuously monitored across four developmental stages (Figure 2). The results showed that internal air temperature consistently exceeded external temperature, with values surpassing 30 °C between 10:00 and 17:00 during the emergence, tuber formation, and starch accumulation stages, indicating the presence of mild heat stress. In addition, soil temperatures within the greenhouse increased significantly during the tuber formation and starch accumulation stages, reaching up to 32 °C at midday. Compared with bare soil (S0), maize stover mulching effectively moderated soil temperature fluctuations—reducing surface overheating during the day and conserving heat at night. Overall, potatoes grown under protected cultivation were subjected to moderate thermal stress, particularly during midday hours. Stover mulching played a beneficial role in stabilizing the soil thermal environment, thus providing a more favorable microclimate for potato growth.

#### 3.1.2 Photosynthetic characteristics and antioxidant capacity

Management strategies significantly altered leaf physiological status. The F2S2 treatment (medium-intensity fertilization with moderate stover mulching) consistently exhibited the highest photosynthetic performance, with the highest chlorophyll content (SPAD value) and net photosynthetic rate (Pn) increasing by 23.5 and 29.4% (Supplementary Figure 1; Supplementary Tables S3–S7), respectively, compared to the control. This was supported by improved photochemical efficiency (Fv/Fm and Y(II)) and reduced thermal dissipation. Furthermore, F2S2 effectively enhanced antioxidant defense, increasing superoxide dismutase (SOD) and catalase (CAT) activities by up to 36.2 and 31.7%, respectively, while malondialdehyde (MDA) content decreased by 21.5% (*p* < 0.05) (Supplementary Figure 2), demonstrating its role in mitigating oxidative stress under protected cultivation.

#### 3.1.3 Soil nutrient characteristics

Stover mulching and fertilization interactively reshaped soil fertility. Moderate mulching (S2) promoted carbon sequestration, with F1S2 (high-intensity) and F2S2 (medium-intensity) showing the most pronounced increases in soil organic matter (up to 19.41 and 12.46%). Notably, the F3S2 treatment (low-intensity fertilization with moderate mulching) exhibited a robust nutrient accumulation profile, significantly enhancing available phosphorus and readily available potassium (increasing by 42.58%) compared to no-mulch controls (Supplementary Figure 3). Low-intensity fertilization (F3) combined with low (S1) or moderate (S2) mulching also strengthened soil nitrogen supply capacity, creating a favorable nutrient reservoir during the tuber bulking stage.

#### 3.1.4 Yield and tuber quality

Integrated practices markedly influenced productivity and quality. While F2S2 was effective in promoting vegetative growth and individual tuber weight, the F3S2 treatment achieved the highest agronomic productivity, with a total yield of 42.33 t·hm^−2^ and a 58.66% increase in tuber weight per plant compared to the control (Supplementary Figure 4; Supplementary Tables S8–S11). Quality-wise, full topdressing combined with moderate mulching (F3S1/F3S2) maximized starch and vitamin C contents, while excessive mulching without fertilization (F0S3) led to undesirable reducing sugar accumulation (Supplementary Figure 5). These findings underscore the effectiveness of moderate (S2) to high (S3) stover mulching combined with dynamic fertilization in balancing yield and commercial quality.

### 3.2 Principal component analysis for identifying the optimal treatment combination

**Figure 3:**
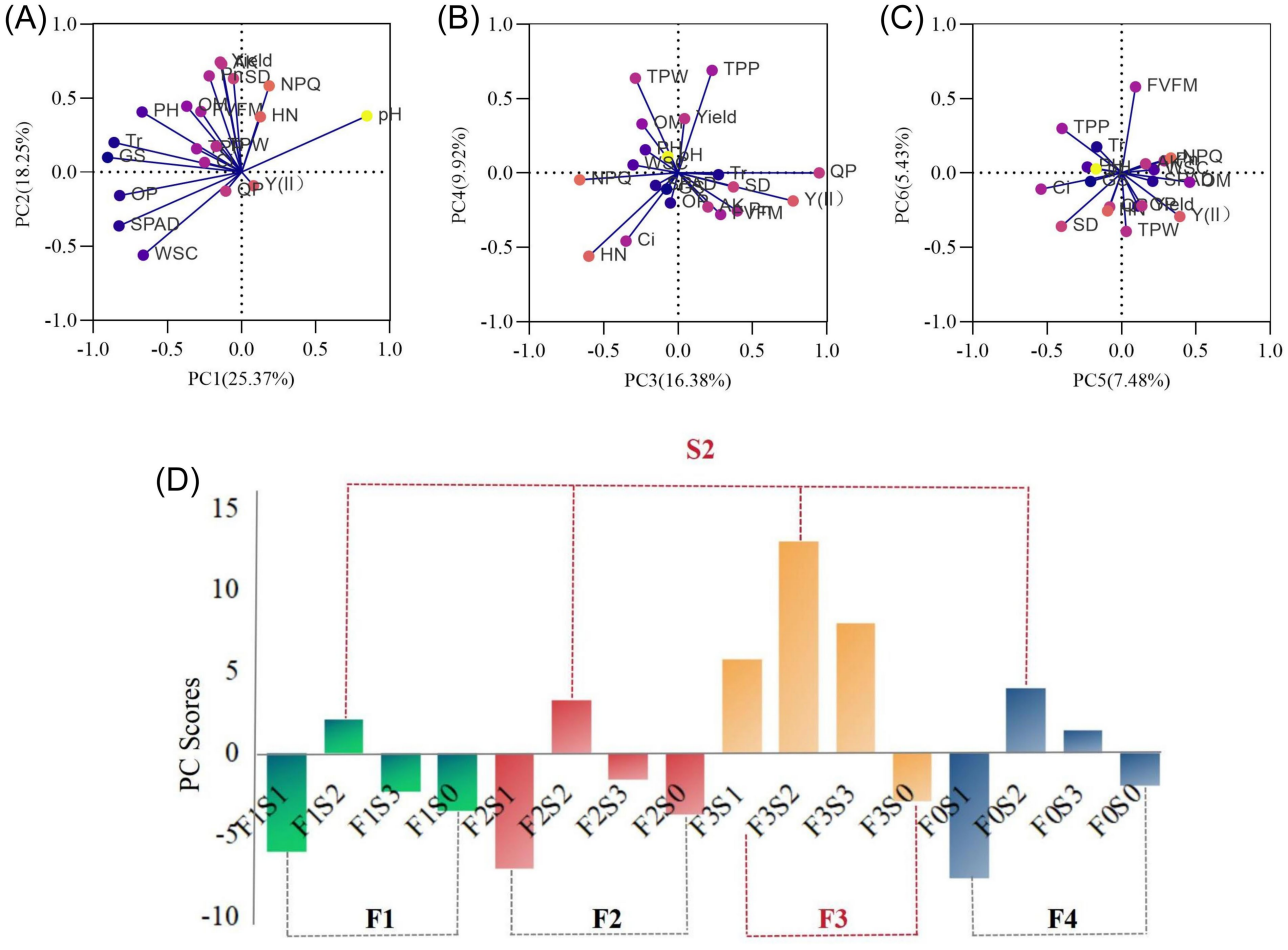
Principal component analysis (PCA) of plant growth, physiological, yield, and quality traits under different stover mulching and fertilization treatments. **(A–C)** PCA biplots of the first six principal components, showing the relationships among multiple agronomic and physiological parameters. Vectors indicate the contribution and direction of each variable to the principal components. **(D)** PC scores of each treatment group based on the integrated analysis of all variables. Treatments are labeled as FxSy, where Fx represents fertilization levels (F0–F3) and Sy indicates stover mulching levels (S0–S3; where S2 is defined as moderate mulching).

To synthesize the multidimensional responses of potato to management regimes, PCA was performed on 21 agronomic and physiological traits (Supplementary Table S12). Seven components were extracted, with the first three (PC1–PC3) explaining 60.0% of the total variance (Supplementary Tables S13, S14). Based on integrated scores, treatment F3S2 obtained the highest comprehensive ranking (12.961), reflecting superior coordination between soil nutrients, plant physiology, and productivity (Figure 3), followed by F3S3 (7.956) and F3S1 (5.741). While certain treatments like F2S2 excelled in specific physiological traits, F3S2 demonstrated the best overall systemic performance. To reveal the biological mechanisms driving these integrated phenotypic outcomes, the rhizosphere microbial community structure and functions under the high-performing F3 fertilization framework were further investigated via metagenomic sequencing.

### 3.3 Variations in soil microbial community structure

To examine the effects of stover mulching intensity on rhizosphere microbial community structure, metagenomic sequencing was performed on soil samples collected at the maturity stage from four stover mulching treatments within the F3 fertilization group, including F3S0 (0 kg·hm^−2^), F3S1 (4,250 kg·hm^−2^), F3S2 (8,500 kg·hm^−2^), F3S3 (17,000 kg·hm^−2^) (Supplementary Figure S6; Supplementary Table S15). Microbial community composition was analyzed at multiple taxonomic levels, together with α- and β-diversity metrics. Sample groups were designated as follows: F3S0 (samples 241–243), F3S1 (211–213), F3S2 (221–223), F3S3 (231–233).

**Figure 4:**
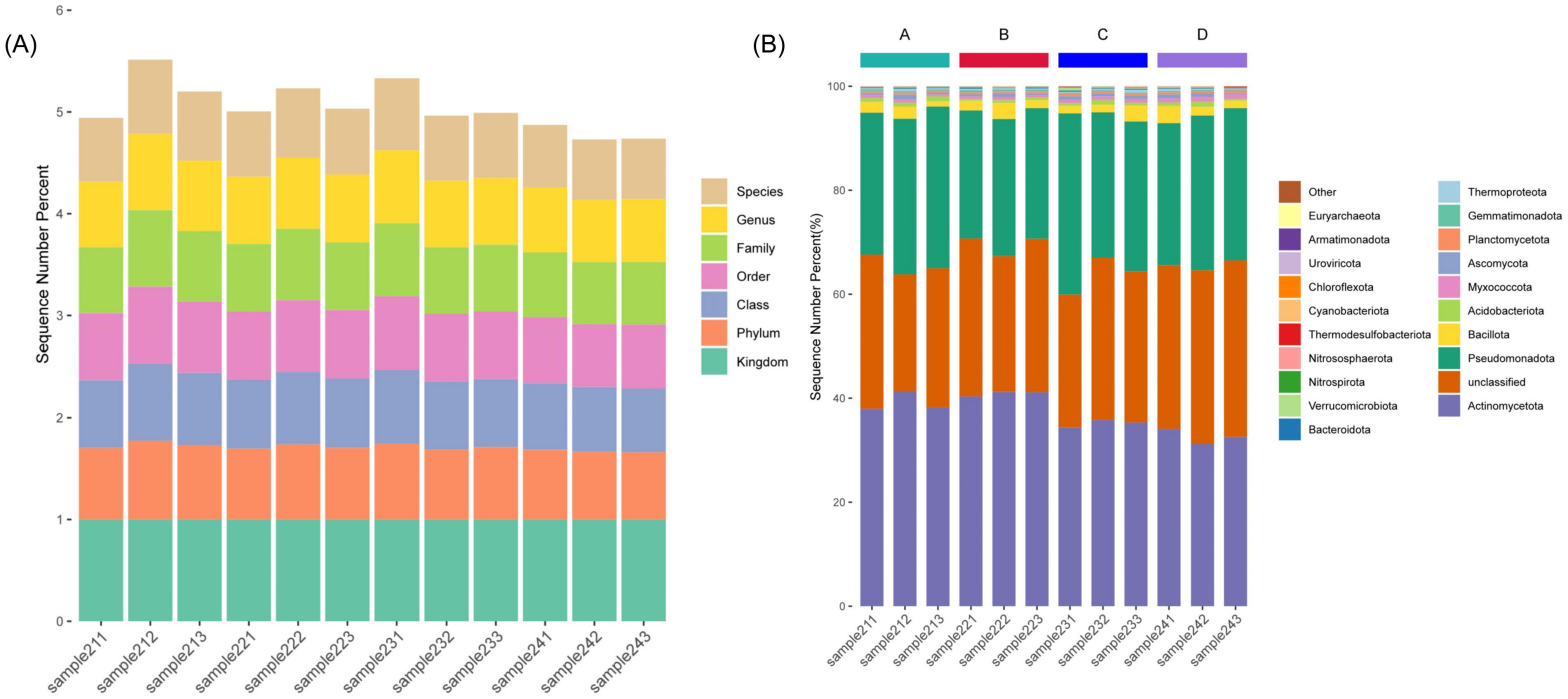
Taxonomic annotation and differential microbial analysis of rhizosphere soil microbiota under different treatments. **(A)** Bar chart showing the annotation depth of each sample across seven taxonomic levels (Kingdom, Phylum, Class, Order, Family, Genus, Species). The *x*-axis indicates sample names, and the *y*-axis shows the proportion of sequences annotated at each taxonomic level. Stacked colors represent different levels according to the legend. **(B)** Relative abundance of dominant phyla in each sample. Only the top 20 phyla are shown for clarity. Colors correspond to phylum names listed in the legend.

Across all samples, Bacteria dominated the microbial community, accounting for 98.53% of total sequences, followed by Archaea (0.79%), Fungi (0.65%), and Viruses (0.01%) (Supplementary Table S16). At the phylum level, the major bacterial taxa included Actinobacteriota, Proteobacteria, Chloroflexi, Acidobacteriota, and Firmicutes (Figure 4A; Supplementary Table S17). The relative abundance of copiotrophic groups, such as Actinobacteriota, reached its highest proportion in the F3S2 treatment (Figure 4B). In contrast, oligotrophic taxa like Acidobacteriota exhibited relatively lower representation in nutrient-rich treatments compared to the no-mulching control.

Hierarchical clustering revealed that samples from F3S2 (moderate) and F3S3 (high) exhibited higher compositional similarity, whereas F3S0 formed a distinct cluster (Supplementary Figure S7). Alpha diversity analysis showed that the Shannon and Simpson indices were significantly higher in F3S2 compared with other treatments, while the lowest values were observed in F3S0. Beta diversity analysis (PCoA) demonstrated a clear separation between mulched and non-mulched treatments, with F3S2 and F3S3 clustering closely, reflecting pronounced differences in community composition associated with stover mulching intensity. Overall, variations in stover mulching rate under optimized fertilization were associated with marked differences in diversity, with moderate (F3S2) and high (F3S3) mulching levels exhibiting higher diversity indices compared to the no-mulching treatment.

### 3.4 Functional annotation of soil microbial communities

Functional annotation was performed using metagenomic data from four stover mulching treatments within the optimized fertilization group (F3S0–F3S3).

#### 3.4.1 KEGG functional annotation

**Figure 5:**
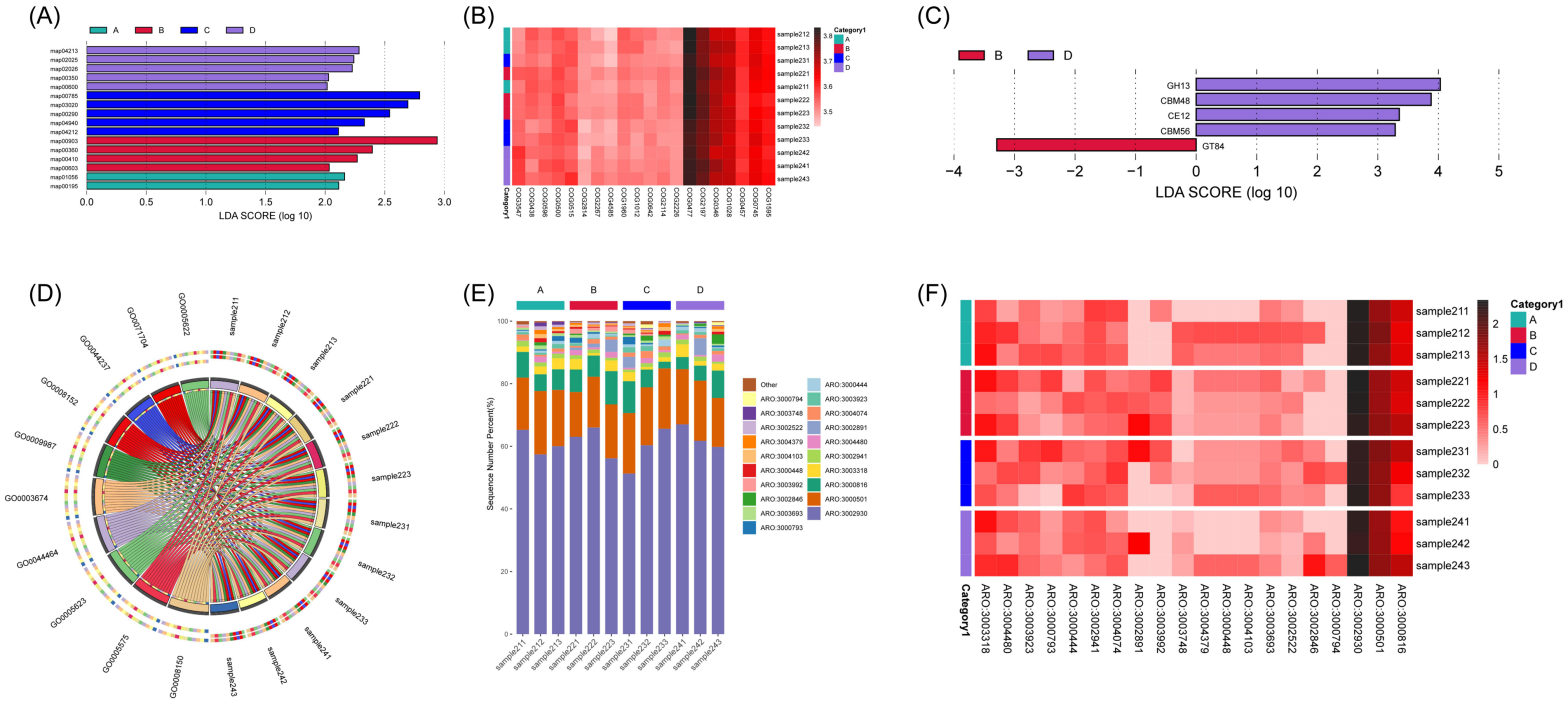
Functional annotation and differential analysis of microbial communities based on metagenomic sequencing. **(A)** LDA bar chart of KEGG metabolic pathways identified by LEfSe analysis (LDA score > 2.0). Each horizontal bar represents a significantly enriched pathway, with bar length corresponding to the LDA score (log_10_). Different colors indicate which treatment group the pathway is characteristic of. **(B)** Clustered heatmap of the top COG categories across all samples. Rows represent samples (colored by treatment groups), and columns represent COG categories, both clustered using Euclidean distance. **(C)** LDA bar chart of carbohydrate-active enzymes (CAZymes) identified via LEfSe analysis. Each bar corresponds to a specific CAZy family, with LDA scores (LDA score > 2.0) representing the magnitude of differential abundance. Colors denote the treatment group in which the CAZyme is most enriched. **(D)** Circos plot showing the distribution of the top 10 most abundant GO terms across all samples. The left half represents individual GO terms, with colors indicating their proportion from each sample; the right half shows sample composition, with different colors representing the corresponding GO terms. **(E)** Relative abundance of the top 20 antibiotic resistance genes (ARGs) across all samples. Less abundant genes are grouped under “Other.” Colors correspond to specific ARG IDs listed in the legend. **(F)** Heatmap showing hierarchical clustering of the top 20 most abundant ARGs. Rows indicate samples, and columns represent ARGs, clustered based on Euclidean distance. Treatment groups are annotated with colored bars on the left.

Genes were predominantly assigned to six level-1 categories, with Metabolism accounting for >70% across all treatments (Supplementary Figure S8). LEfSe analysis at KEGG level-3 revealed that the F3S2 (moderate) treatment showed higher enrichment in pathways related to carbon metabolism (map00600), the pentose phosphate pathway (map00030), and membrane transport (map02026) (Figure 5A; Supplementary Table S18). The enrichment of carbon metabolism in F3S2 is highly consistent with the observed increase in Actinobacteriota, suggesting this phylum is a primary contributor to enhanced carbon cycling in the rhizosphere. In contrast, F3S3 (high) was characterized by enrichment in nitrogen metabolism (map00910).

#### 3.4.2 eggNOG (COG) and GO functional annotation

Differences were primarily observed in COG categories related to energy production (C), amino acid transport (E), and carbohydrate transport (G) (Figure 5B; Supplementary Table S19). F3S2 displayed higher relative abundance in carbohydrate transport and metabolism, aligning with the high metabolic activity of the enriched Actinobacteriota population.

Compared with other treatments, F3S2 samples showed relatively higher associations with several metabolism-related GO terms (Figure 5D; Supplementary Table S20).

#### 3.4.3 CAZy functional annotation

Annotation of carbohydrate-active enzymes (CAZymes) revealed that F3S2 exhibited higher relative abundance of families including GH13, CBM48/CBM56, and CE12, involved in carbohydrate metabolism (Figure 5C; Supplementary Table S21). This enrichment is closely linked to the dominance of Actinobacteriota, which possess a diverse repertoire of enzymes for the degradation of complex organic matter like maize stover.

Correlation analysis showed that the relative abundance of copiotrophic Actinobacteriota exhibited a strong positive correlation with soil organic phosphorus (*r* = 0.623, *p* < 0.05). The highest abundance of Actinobacteriota (~41%) in the F3S2 treatment coincided with significantly elevated OP levels (1,324 mg·kg^−1^) compared to the control. In contrast, oligotrophic Acidobacteriota showed a negative correlation with nutrient availability (Supplementary Table S22). These results confirm that the enrichment of specific rhizosphere functions is primarily driven by soil nutrient redistribution under moderate stover mulching.

### 3.5 Antibiotic resistance gene (ARG) annotation

To provide a comprehensive assessment of the environmental biosafety and ecological stability of the stover-amended rhizosphere system, metagenomic data from four low-intensity (full topdressing) fertilization treatments (F3S0–F3S3) were annotated against the CARD database (Supplementary Table S23).

Analysis of relative abundance revealed that several ARGs were consistently detected across all treatments, including *ARO:3002522*, *ARO:300479*, *ARO:3000794*, *ARO:3000744*, and *ARO:3000501* (Figure 5E). These ARGs are associated with diverse resistance mechanisms such as efflux pumps, target modification, and enzymatic inactivation. Among the treatments, F3S2 (moderate mulching) and F3S3 (high mulching) exhibited a broader ARG composition, with multiple ARGs showing comparatively higher relative abundance. Notably, *ARO:3002522*, which encodes an antibiotic efflux protein, was more abundant in the F3S2 treatment.

Hierarchical clustering based on ARG abundance profiles showed that samples within the F3S2 treatment clustered closely, indicating relatively consistent ARG composition among replicates (Figure 5F). In contrast, samples from the no-mulching treatment (F3S0) were more dispersed and generally exhibited lower ARG abundance. The Circos diagram (Supplementary Figure S9) further illustrated the associations between samples and high-abundance ARGs. F3S2 samples were linked to a greater number of ARG types, whereas fewer ARGs were prominently associated with F3S0 samples. These results indicate that maize stover mulching intensity was associated with differences in ARG composition and distribution under optimized fertilization conditions. These observations suggest that the optimized management strategy (F3S2) potentially shapes a more complex microbial interaction network, where the enrichment of specific ARGs may be linked to the increased abundance of antibiotic-producing taxa such as Actinobacteriota. This recruitment reflects a reshaped ecological niche characterized by intensified microbial competition and resource-driven taxonomic shifts.

## 4 Discussion

### 4.1 Synergistic effects of stover mulching and fertilization on potato physiology

This study demonstrates that integrated maize stover mulching and fertilization practices modify the greenhouse soil–plant environment, thereby influencing potato physiological performance. Rather than conferring uniform benefits, the observed responses were highly dependent on the fertilization strategy and mulching intensity, highlighting the importance of coordinated management.

Soil temperature regulation is critical for potato tuber development, particularly in greenhouses where midday heat stress often exceeds the optimal range of 16–18 °C (Haverkort and Verhagen, 2008; Shaodong et al., 2022). Our results show that moderate stover mulching (S2) buffered diurnal soil temperature fluctuations, mitigating surface overheating during the day. This thermal moderation aligns with reports that stover mulch stabilizes the rhizosphere microclimate by balancing insulation and heat dissipation (Liu et al., 2021).

In addition to thermal effects, stover mulching combined with optimized fertilization influenced vegetative growth. While direct soil moisture was not monitored in this study, the potential for moisture conservation and improved soil aeration under moderate mulching—as suggested by previous literature—likely facilitated root development and nutrient uptake. Aligning staged fertilization with crop demand further enhanced these growth traits, consistent with findings in other integrated organic–inorganic systems (Xing et al., 2022; Du et al., 2022).

Photosynthetic performance and redox regulation were also sensitive to these practices. Treatments with moderate mulching and balanced fertilization (e.g., F2S2) exhibited superior chlorophyll content and photochemical efficiency. These responses are likely associated with stabilized nitrogen supply and optimized microenvironmental conditions, which support photosystem stability (Garnett et al., 2009; Liu et al., 2019). Furthermore, enhanced antioxidant enzyme activities (SOD, CAT) and reduced MDA content suggest improved oxidative balance, supporting the role of stover-amended systems in stress mitigation (Patil Shirish et al., 2013; Song et al., 2024). Overall, stover mulching and fertilization interact to influence potato performance primarily through improved thermal stability, nutrient availability, and oxidative stress reduction.

### 4.2 Coordinated effects of stover mulching and fertilization on soil nutrient status

In the present study, stover mulching and fertilization strategies jointly reshaped soil nutrient availability. It is noteworthy that the F3S2 combination (low-intensity, 100% topdressing fertilization with moderate mulching) achieved the highest productivity despite receiving lower total nutrient inputs compared to high-intensity basal treatments (F1).

The F3S2 treatment was associated with significantly higher soil organic matter, available phosphorus, and potassium. This suggests that moderate organic input combined with dynamic nutrient supply (topdressing) enhances nutrient retention in the rhizosphere. The increase in soil organic matter under moderate mulching is attributed to carbon inputs from stover decomposition, which contributes to long-term soil carbon cycling (Liu J. et al., 2024). Concurrently, concentrating nutrient inputs during the tuber bulking stage via topdressing aligned with periods of peak crop demand, while stover mulching likely reduced potential nutrient losses.

Available phosphorus levels were also modulated by mulching intensity, indicating that stover-derived organic inputs can influence P availability by altering soil organic matter content and rhizosphere chemical conditions (Li et al., 2015; Waheed et al., 2023). Notably, phosphorus responses varied even under identical fertilization regimes, underscoring the independent regulatory role of maize stover mulching. These results indicate that moderate stover mulching combined with optimized topdressing fertilization promotes a favorable nutrient reservoir that supports yield formation under intensive greenhouse cultivation.

### 4.3 Synergistic effects of stover mulching and fertilization on potato yield and quality

The present study indicates that stover mulching and fertilization strategies jointly influenced potato yield components and tuber quality. Among the treatments, the combination of low-intensity fertilization (full topdressing) with moderate stover mulching (F3S2) exhibited the most favorable coordination between yield and quality.

In terms of yield, F3S2 increased total yield by 42.3% compared to the control. These responses are likely linked to the combined effects of stover mulching and topdressing, which stabilize the soil microenvironment and align nutrient availability with crop demand during the tuber bulking stage. The potential for reduced soil moisture loss and buffered temperature fluctuations under stover mulching—as reported in previous studies (Ferdoushi et al., 2010; Souza et al., 2020; Ravichandran et al., 2022)—likely supported tuber expansion.

Quality-related traits also responded to integrated management. Under high mulching without fertilization (F0S3), higher reducing sugar accumulation was observed, likely due to delayed maturation in cooler soils. In contrast, F3S2 maintained higher Vitamin C content, possibly due to optimized potassium availability during late growth stages (El-Hadidi et al., 2017; Zhang et al., 2022). Notably, no trade-off between yield and quality was observed under F3S2, suggesting that coordinating mulching intensity with fertilization timing supports both productivity and commercial value (Ju et al., 2024).

### 4.4 Microbial community structure and functional characteristics

Soil microbial communities are essential regulators of nutrient cycling (Rajguru et al., 2024). Using metagenomic sequencing, we examined how maize stover mulching combined with low-intensity fertilization influenced the rhizosphere (Nwachukwu and Babalola, 2022). Importantly, our sampling at the maturity stage was designed to capture the cumulative long-term effects and the “soil legacy” shaped by these management practices throughout the growing season.

Bacteria dominated all treatments, with Actinobacteriota, Proteobacteria, Chloroflexi, and Acidobacteriota representing the major phyla (Piao et al., 2008). Moderate stover mulching (F3S2) significantly increased α-diversity and reshaped community structure. This shift is tied to the high-resource niche created by stover decomposition and targeted fertilization, which recruited copiotrophic microorganisms. Copiotrophs, such as Actinobacteriota and Proteobacteria, are characterized by high metabolic activity in nutrient-rich environments. Conversely, oligotrophic groups like Acidobacteriota declined in relative abundance, reflecting a competitive shift in resource-abundant conditions. Correlation analysis confirmed this, as Actinobacteriota abundance was positively linked to organic phosphorus (*r* = 0.623, *p* < 0.05), explaining why the F3S2 treatment promoted a more specialized functional community (Banerjee et al., 2019).

Functional annotation revealed that genes related to carbon metabolism and carbohydrate processing were enriched in the F3S2 treatment. This was supported by the higher abundance of carbohydrate-active enzyme (CAZyme) families such as GH13, CBM48, and CE12, which are involved in polysaccharide degradation (Dou et al., 2023). These shifts indicate a higher potential for stover-derived carbon processing (Lombard et al., 2014; Drula et al., 2022; Omotayo et al., 2022). The dominance of Actinobacteriota in F3S2-treated soils likely drives this metabolic potential, as this phylum possesses the complex enzymatic machinery necessary to break down organic matter like maize stover (Liu S. et al., 2024).

Metagenomic analysis revealed a higher abundance and diversity of antibiotic resistance genes (ARGs) in the F3S2 treatment. Rather than indicating an environmental risk, this enrichment reflects an intensified microbial interaction network. The occurrence of ARGs is an intrinsic component of soil microbial communities, often shaped by interspecific competition (Willms et al., 2021; Wu et al., 2023; Wang W. et al., 2023). In our study, the higher ARG diversity under F3S2 likely reflects the increased recruitment of antibiotic-producing taxa, such as Actinobacteriota, and the subsequent competitive pressure within the resource-rich rhizosphere. Such conditions favor microbial coexistence and functional diversification, where resistance traits support survival under high-density competitive conditions (Martínez, 2008; Aminov, 2009). Therefore, the ARG profile serves as a complementary indicator of the complex ecological niche shaped by stover and fertilizer integration.

Overall, this study demonstrates that moderate stover mulching combined with optimized topdressing fertilization provides a superior management option for greenhouse potato. While these findings are derived from a specific cultivar and controlled conditions, they provide a mechanistic understanding of how microbiome-informed strategies can support sustainable intensification in protected cultivation.

## Data Availability

The datasets generated for this study can be found in the NCBI SRA database at: https://www.ncbi.nlm.nih.gov/, PRJNA1271939.
