# Supplementary material for: Maize stover mulching combined with an optimized fertilization strategy reshapes rhizosphere microbial communities and functions in greenhouse potato
Source: Front Microbiol. 2026 May 5;17:1670904. doi: 10.3389/fmicb.2026.1670904 (PMC13183800; doi:10.3389/fmicb.2026.1670904)
Supplement: Supplementary file 1 [file Data_Sheet_1.ZIP › Supplementary_Material/Supplementary_Material.docx]

Supplementary Material

# Supplementary Figures
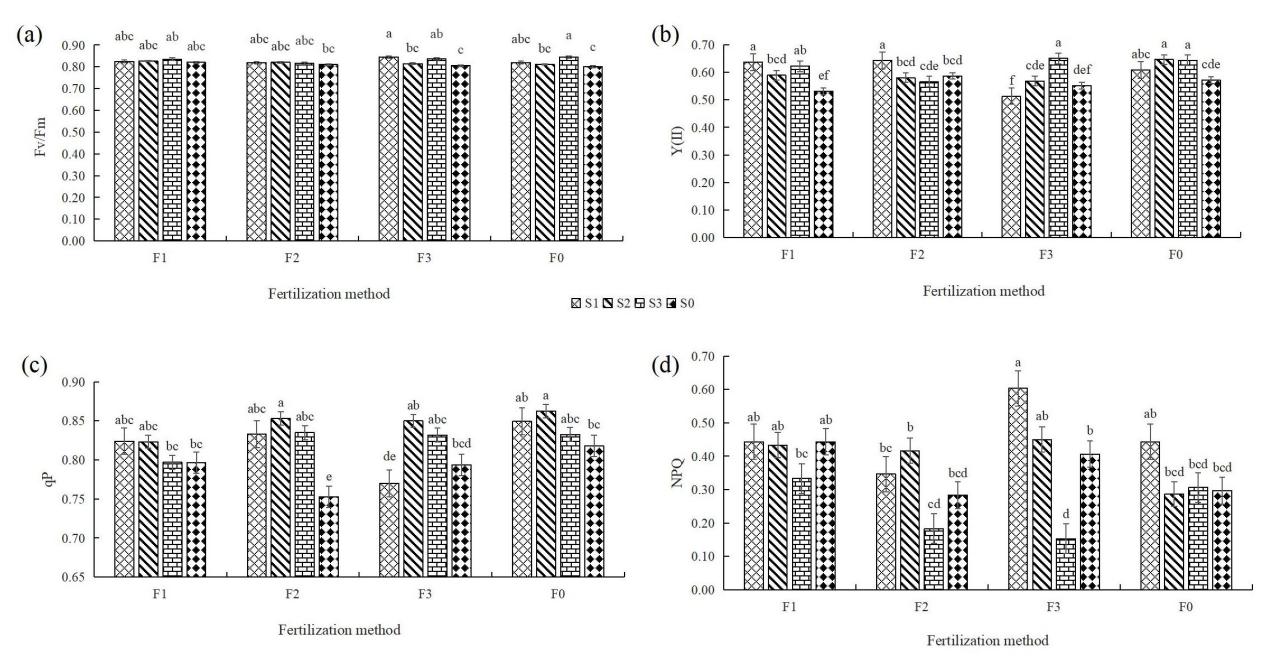


# **Supplementary Figure 1**. Effects of different straw mulching and fertilization combinations on photosynthetic fluorescence parameters of potato leaves at the tuber expansion stage. (a) Fv/Fm: maximum photochemical efficiency of PSII. (b) Y(II): effective quantum yield of PSII. (c) qP: photochemical quenching coefficient. (d) NPQ: non-photochemical quenching coefficient. F1–F3 represent three fertilization regimes: F1 (basal fertilizer only), F2 (half basal + half topdressing), F3 (full topdressing); F0 represents the unfertilized control. S0–S3 represent four straw mulching levels: S0 (no mulching), S1 (4250 kg·hm⁻²), S2 (8500 kg·hm⁻²), S3 (19000 kg·hm⁻²). Different lowercase letters indicate significant differences between treatments (P < 0.05, Duncan’s multiple range test).
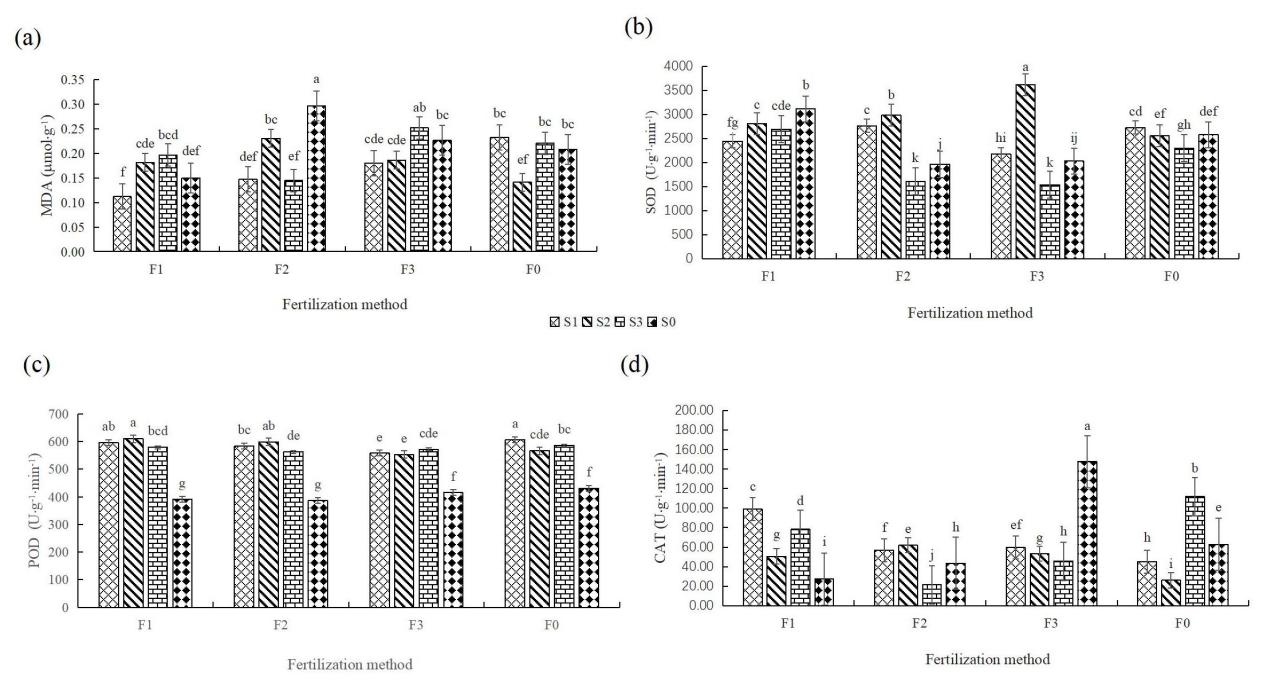


**Supplementary Figure 2.** Effects of different straw mulching and fertilization combinations on antioxidant enzyme activity and lipid peroxidation in potato leaves at the tuber expansion stage. (a) MDA: malondialdehyde content. (b) SOD: superoxide dismutase activity. (c) POD: peroxidase activity. (d) CAT: catalase activity. F1–F3 represent three fertilization regimes: F1 (basal fertilizer only), F2 (half basal + half topdressing), F3 (full topdressing); F0 represents the unfertilized control. S0–S3 represent four straw mulching levels: S0 (no mulching), S1 (4250 kg·hm⁻²), S2 (8500 kg·hm⁻²), S3 (19000 kg·hm⁻²). Different lowercase letters above bars indicate significant differences between treatments (P < 0.05, Duncan’s multiple range test). Error bars represent standard deviation (n = 3).


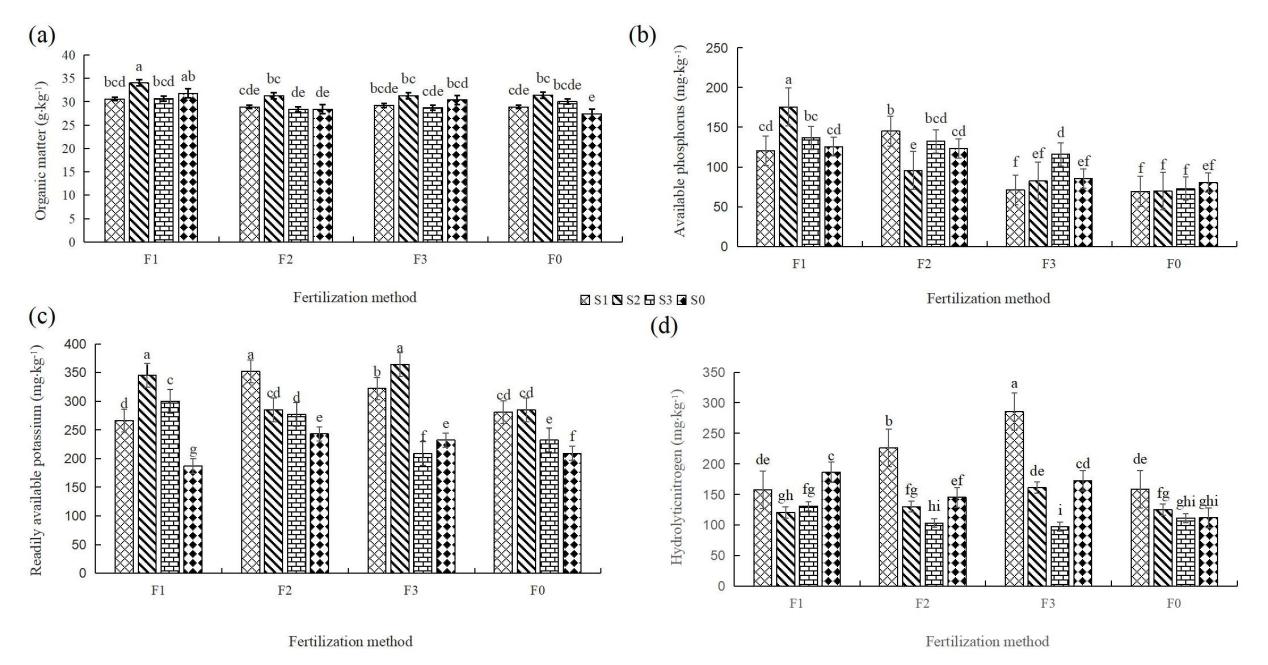


**Supplementary Figure 3.** Effects of different straw mulching and fertilization combinations on soil physicochemical properties at the potato tuber expansion stage. (a) Soil organic matter content (g·kg⁻¹). (b) Available phosphorus (mg·kg⁻¹). (c) Readily available potassium (mg·kg⁻¹). (d) Hydrolyzable nitrogen (mg·kg⁻¹). F1–F3 represent three fertilization regimes: F1 (basal fertilizer only), F2 (half basal + half topdressing), F3 (full topdressing); F0 represents the unfertilized control. S0–S3 represent four straw mulching levels: S0 (no mulching), S1 (4250 kg·hm⁻²), S2 (8500 kg·hm⁻²), S3 (19000 kg·hm⁻²). Different lowercase letters indicate significant differences among treatments at P < 0.05 (Duncan’s test). Values are means ± SD (n = 3).


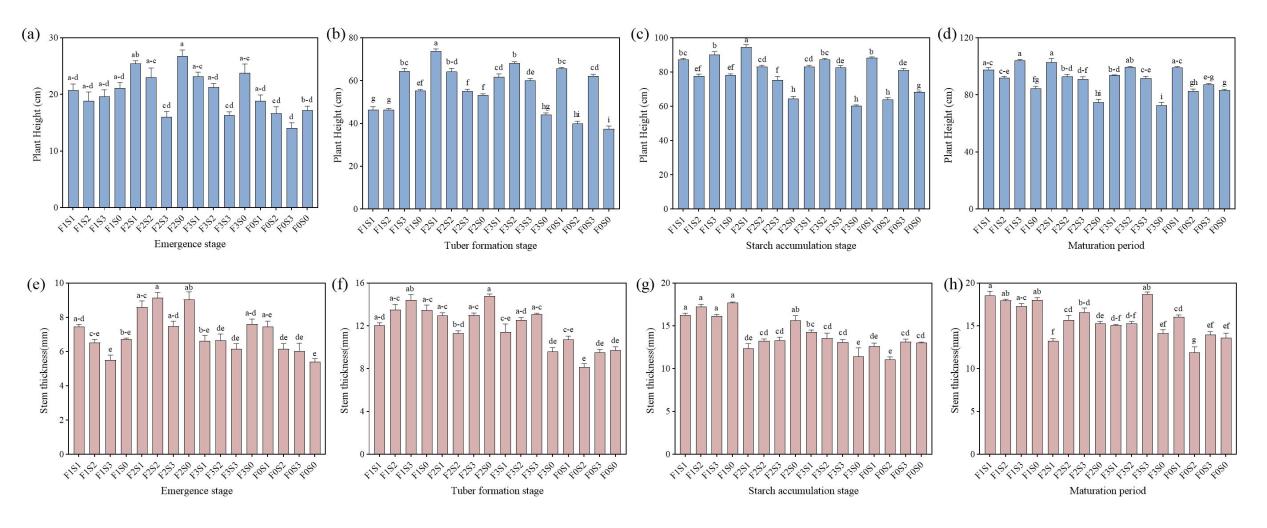


**Supplementary Figure 4.** Effects of different straw mulching and fertilization combinations on potato plant height and stem diameter at various growth stages. (a–d) Plant height (cm) measured at the emergence stage, tuber formation stage, starch accumulation stage, and maturation period, respectively. (e–h) Stem diameter (mm) measured at the corresponding growth stages. Treatments are denoted as combinations of fertilization regimes (F1: basal fertilizer only, F2: 50% basal + 50% topdressing, F3: full topdressing, F0: no fertilization) and straw mulching levels (S0: no straw, S1: 4250 kg·hm⁻², S2: 8500 kg·hm⁻², S3: 19000 kg·hm⁻²). Different lowercase letters indicate significant differences among treatments at P < 0.05 based on Duncan’s multiple range test. Values are means ± SD (n = 3).


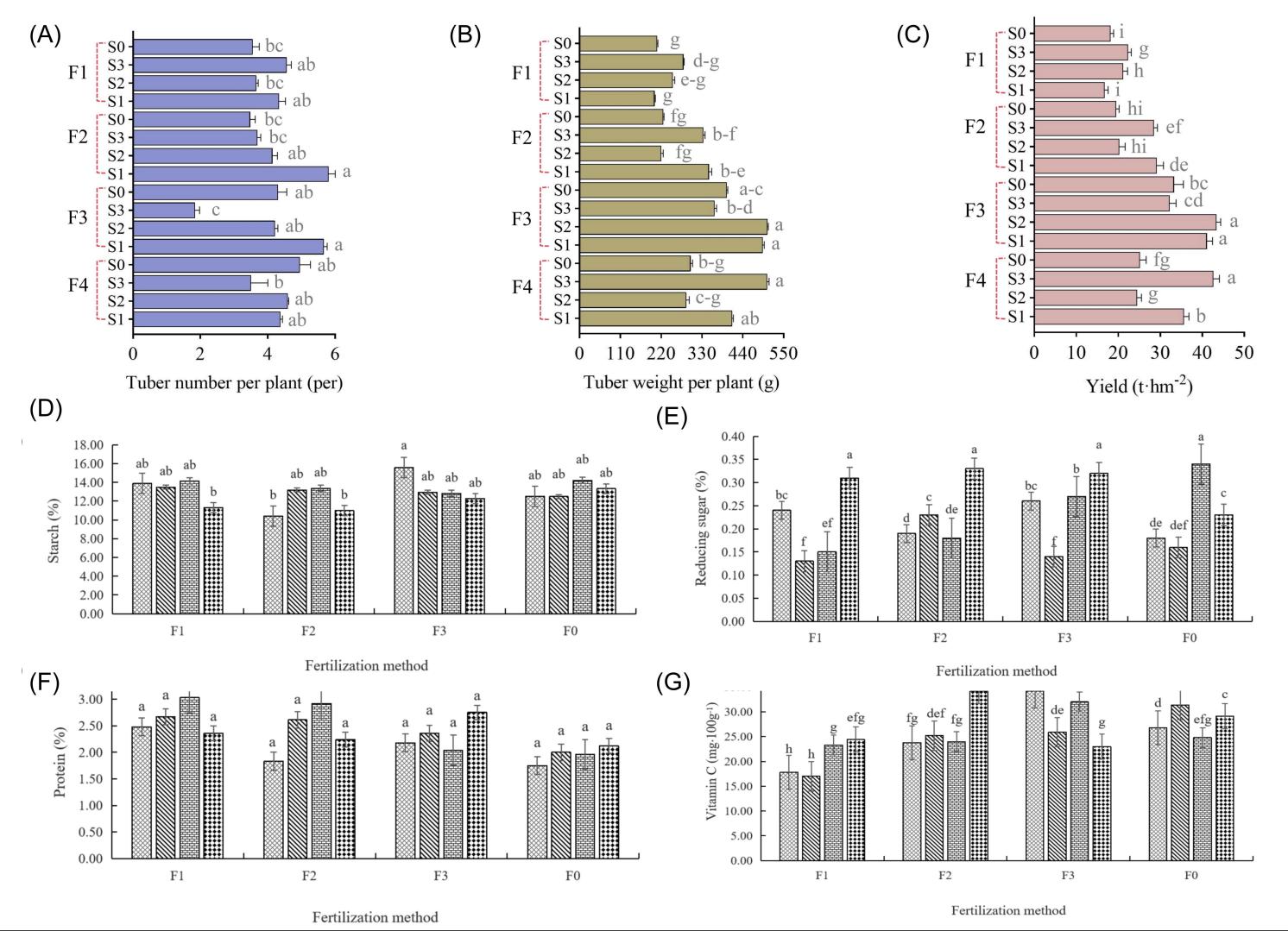


**Supplementary Figure 5.** Effects of straw mulching and fertilization treatments on potato yield components and quality traits. (A–C) Effects on tuber number per plant (A), tuber weight per plant (B), and yield per hectare (C) under different combinations of fertilization (F1–F4) and straw coverage levels (S0–S3). (D–G) Effects on key quality traits: starch content (D), reducing sugar content (E), protein content (F), and vitamin C content (G). Different lowercase letters indicate significant differences between treatments at p < 0.05 according to LSD test. Treatments are coded as FxSy, where Fx denotes fertilization level and Sy indicates straw coverage level.


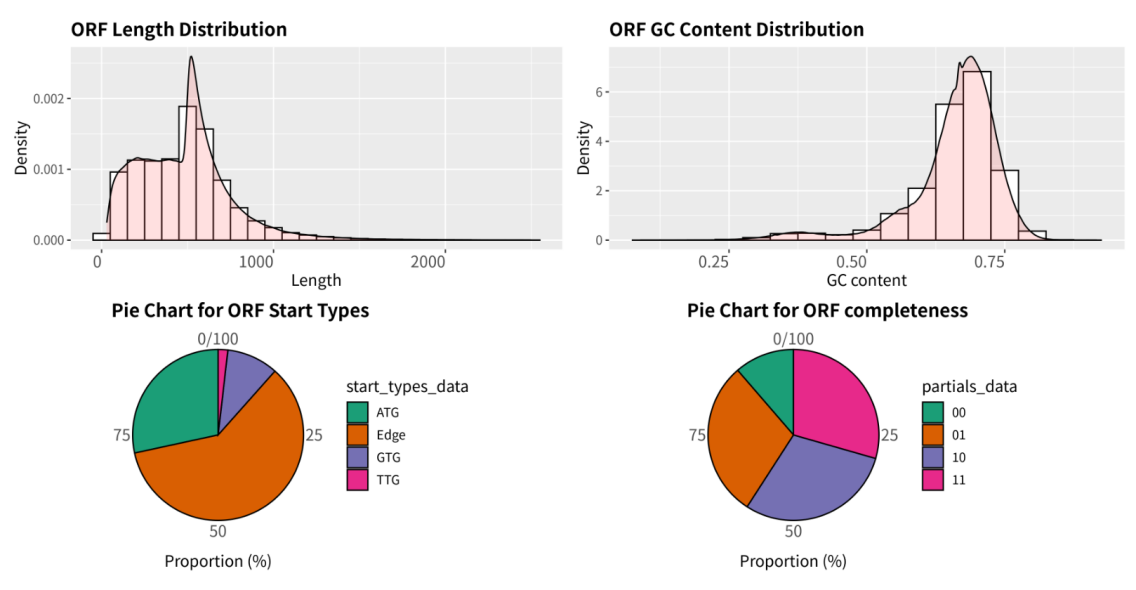


**Supplementary Figure 6.** Summary statistics of predicted genes from metagenomic data. (Top left) Histogram of open reading frame (ORF) length distribution, reflecting the overall coding sequence length characteristics. (Top right) Histogram of GC content distribution among predicted ORFs. (Bottom left) Pie chart of predicted ORF start codon types, including standard start codons (ATG, GTG, TTG) and “Edge” for undetermined start positions. (Bottom right) Pie chart of ORF completeness based on presence of start and stop codons: “00” indicates complete ORFs (with both start and stop codons), “01” indicates ORFs with only a start codon, “10” indicates ORFs with only a stop codon, and “11” indicates ORFs lacking both.


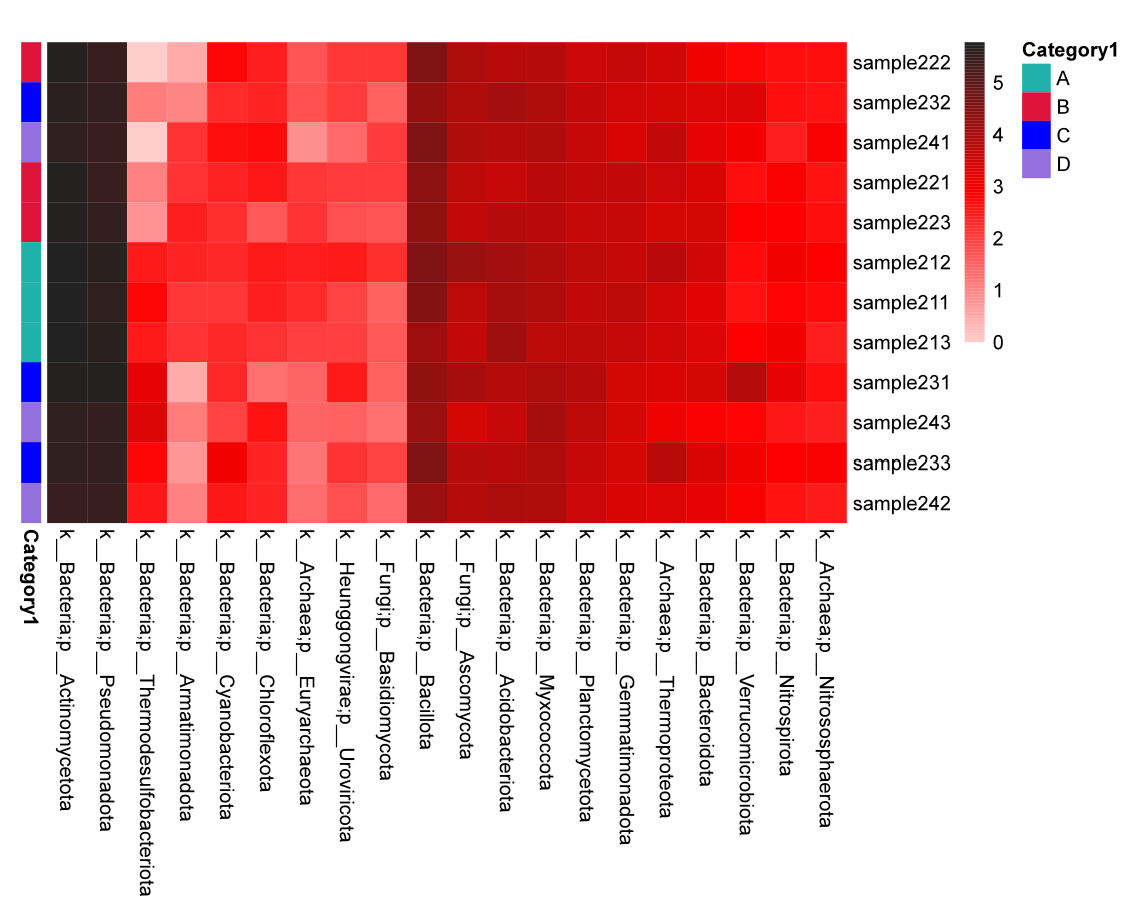


**Supplementary Figure 7.** Hierarchical clustering heatmap of microbial communities at the phylum level. The horizontal axis represents microbial taxa annotated at the phylum level, while the vertical axis shows individual samples, labeled by treatment groups (A: F3S1, B: F3S2, C: F3S3, D: F3S4). The heatmap color gradient reflects the relative abundance of each taxon in each sample, as indicated by the color scale bar. The dendrogram at the top shows clustering based on taxonomic similarity across samples, while the dendrogram on the left reflects clustering of samples based on community composition.


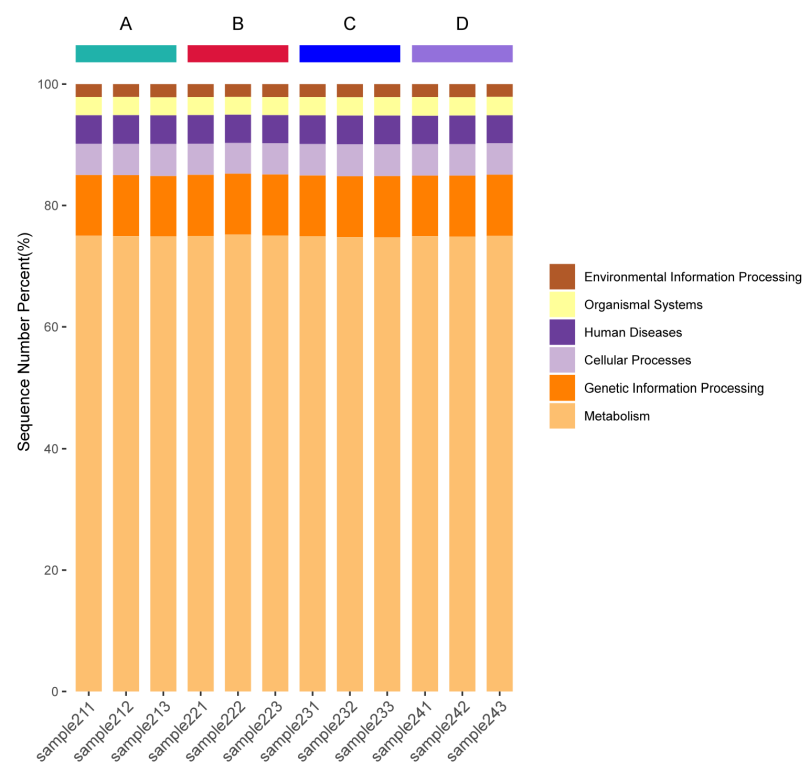


**Supplementary Figure 8.** Bar chart showing the relative abundance of KEGG metabolic pathways at Level 1. Each bar represents the functional composition of a single sample based on KEGG orthologous group annotation. Colors indicate the six major KEGG Level 1 functional categories: Metabolism, Genetic Information Processing, Cellular Processes, Human Diseases, Organismal Systems, and Environmental Information Processing. Sample groups A–D correspond to treatments F3S1 to F3S4, respectively.


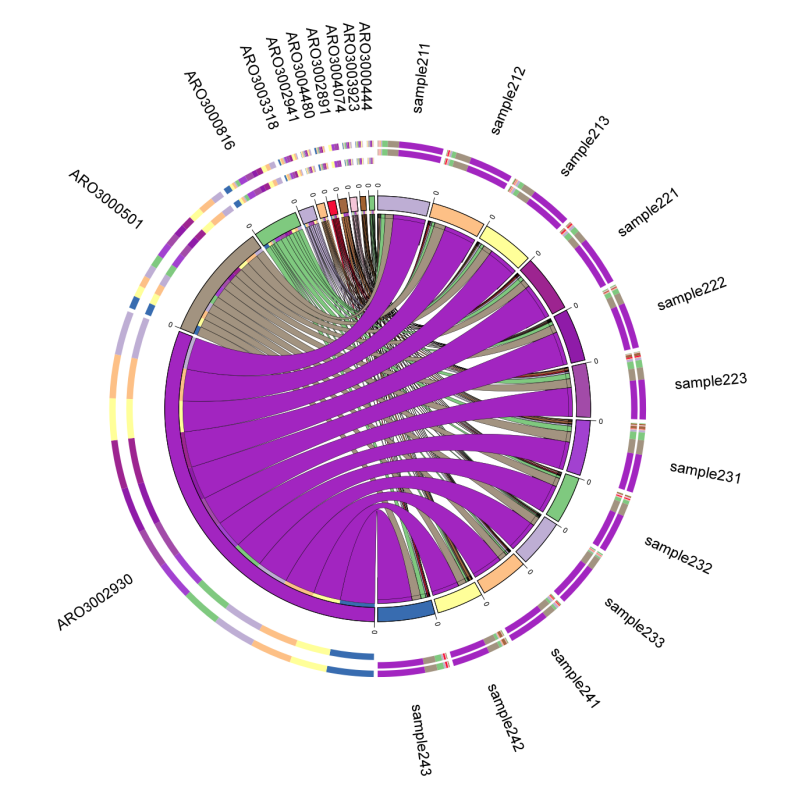


**Supplementary Figure 9.** Circos plot showing the distribution of antibiotic resistance genes (ARGs) across different soil samples. The left semicircle represents the top 10 ARGs with the highest relative abundance, where the color segments indicate the proportional contributions from each sample. The right semicircle displays all analyzed soil samples (sample211–sample243), with internal color bands representing the relative composition of each ARG within the corresponding sample. This visualization highlights the diversity and distribution patterns of key ARGs among different straw mulching treatments.
